# Prevalence and Risk Factors for Anti-SARS-CoV-2 Antibody in Chronic Kidney Disease (Dialysis Independent and Not)

**DOI:** 10.3390/pathogens11050572

**Published:** 2022-05-12

**Authors:** Mariana Siddi, Paolo Molinari, Carlo Maria Alfieri, Marianna Tangredi, Giovanna Lunghi, Elisa Colombo, Sara Uceda Renteria, Emanuele Grimaldi, Ferruccio Ceriotti, Giuseppe Castellano, Fabrizio Fabrizi

**Affiliations:** 1 Division of Nephrology, Dialysis and Kidney Transplant, Maggiore Policlinico Hospital and IRCCS Ca’ Granda Foundation, 20122 Milano, Italy; mariana.siddi@policlinico.mi.it (M.S.); paolo.molinari@policlinico.mi.it (P.M.); carlomaria.alfieri@policlinico.mi.it (C.M.A.); marianna.tangredi@policlinico.mi.it (M.T.); elisa.colombo@policlinico.mi.it (E.C.); emanuele.grimaldi@policlinico.mi.it (E.G.); giuseppe.castellano@policlinico.mi.it (G.C.); 2 Department of Clinical Sciences and Community Health, University of Milan, 20122 Milano, Italy; 3 Clinical Laboratory, Maggiore Policlinico Hospital and IRCCS Ca’ Granda Foundation, 20122 Milano, Italy; giovanna.lunghi@policlinico.mi.it (G.L.); sara.uceda@policlinico.mi.it (S.U.R.); furio.ceriotti@pioliclinico.mi.it (F.C.)

**Keywords:** chronic kidney disease, COVID-19, dialysis, epidemiology, SARS-CoV-2, serology

## Abstract

Background: The evidence in the medical literature regarding the prevalence of antibody towards SARS-CoV-2 in patients with chronic kidney disease is limited, particularly among those at the pre-dialysis stage. Aim: We have prospectively performed a cohort study at a third-level university hospital to evaluate frequency and risk factors for anti-SARS-CoV-2-positive serology among chronic kidney disease patients. Methods: We have tested a cohort of consecutive outpatients with chronic kidney disease on regular follow-up at a major metropolitan hospital, during the SARS-CoV-2 outbreak in Italy. We adopted an enzyme immunoassay for the assessment of IgM/IgG antibodies to SARS-CoV-2 in human serum or plasma (DIA.PRO COVID-19 Serological Assay); the assay detects antibodies against Spike (1/2) and Nucleocapsid proteins of the SARS-CoV-2 genome. Results: There were 199 (65.8%) out of 302 patients with dialysis-independent CKD; 2 patients were anti-SARS-CoV-2 IgM antibody positive, 23 were anti-SARS-CoV-2 IgM/IgG positive and 37 had detectable anti-SARS-CoV-2 IgG antibody in serum. The prevalence of anti-SARS-CoV-2 IgG was 20.5% (60/302). All patients positive for anti-SARS-CoV-2 antibody tested negative by nasopharyngeal swab. A significant and independent relationship between anti-SARS-CoV-2-positive serologic status and serum albumin (a marker of nutritional status) was observed (*p <* 0.046). The prevalence of anti-SARS-CoV-2 antibody was greater in CKD than in control populations (health care workers and blood donors) attending the hospital a few months before the current study (7.6% and 5.2%, respectively). Conclusions: The great prevalence of anti-SARS-CoV-2 antibody in our study group could be, at least partially, explained with the fact that our patients were living in Milan, an area severely hit by SARS-CoV-2 infection. It seems that a poor nutritional status supports the acquisition of SARS-CoV-2 antibody in CKD patients. Clinical studies to understand the mechanisms responsible for the high frequency of SARS-CoV-2 infection are under way.

## 1. Introduction

The European Centre for Disease Prevention and Control has reported (since 31 December 2019 and as of week 2022-17) 512,690,034 cases of COVID-19 worldwide (in accordance with the applied case definitions and testing strategies in the affected countries), including 6,252,316 deaths [1]. COVID-19 is a contagious disease caused by severe acute respiratory syndrome coronavirus 2 (SARS-CoV-2); it was originally reported in Wuhan, China, and since then has spread worldwide [2,3]. The World Health Organization (WHO) declared COVID-19 a pandemic in March 2020 [4]. 

The clinical manifestations of infection by SARS-CoV-2 are heterogeneous [5]; COVID-19 patients can be asymptomatic, and it has been calculated that at least a third of people who are infected do not develop noticeable symptoms. Patients with COVID-19 can show mild symptoms of the upper respiratory tract or develop viral pneumonia with respiratory failure and eventually death. Further, multi-organ involvement has been observed in patients with SARS-CoV-2 infection (damage to kidneys, heart and gastrointestinal tract). As an example, acute kidney injury is common in patients with SARS-CoV-2 infection during their hospital stay [6,7] 

Patients with end-stage kidney disease on long-term dialysis have an increased risk of exposure to SARS-CoV-2; they commonly have some putative risk factors for COVID-19 including advanced age, high frequency of comorbidities (arterial hypertension, diabetes mellitus, obesity, among others), or dense urban geographic location [8]. Pre-dialysis or dialysis patients have an impaired immune (cellular and humoral) response conferred from uraemia. 

The death rate in dialysis patients with SARS-CoV-2 infection appears much greater than that reported in those dialysis patients who are not infected. According to recent data from 12,501 patients undergoing maintenance dialysis in Canada, 187 (1.5%) were diagnosed with SARS-CoV-2 infection; the case fatality rate was 28.3% and 5.8% in SARS-CoV-2 infected and non-infected patients on dialysis [9]. 

Serologic testing can be used to monitor the frequency of the disease and to evaluate screening policies or protocols to limit transmission within dialysis facilities. The evidence in the medical literature regarding the epidemiology and clinical significance of antibody towards SARS-CoV-2 in CKD population is very scarce [5]. This study investigates the prevalence and risk factors for detectable anti-SARS-CoV-2 antibody in a cohort of chronic kidney disease patients on regular follow-up at a major hospital of Milan city. The metropolitan area of Milan is located in Lombardy, by far the Italian region most affected by the COVID-19 outbreak. 

## 2. Results

From 10 August 2020 to 15 February 2021, 302 patients with chronic kidney disease provided a blood sample and completed the questionnaire. There were 199 (65.8%) patients with CKD at pre-dialysis stage, and 103 on regular haemodialysis (Table 1). We found 62 (20.5%) patients with positive serology for anti-SARS-CoV-2 antibody, all tested negative by nasopharyngeal swab. Two patients were anti-SARS-CoV-2 IgM antibody positive, 23 were anti-SARS-CoV-2 IgM/IgG positive and 37 had detectable anti-SARS-CoV-2 IgG antibody in serum. Weak positive patients were not considered anti-SARS-CoV-2-positive patients. ELISA testing showed that IgG SARS-CoV-2-positive sera had greater levels of optical density compared with those from SARS-CoV-2-negative patients, 6.14 ± 5.37 vs. 0.67 ± 0.95, *p* = 0.0001. 

Underlying nephropathies were as follows: diabetic nephropathy (*n* = 48), nephroangiosclerosis (*n* = 109), APKD (*n* = 7), obstructive nephropathy (*n* = 10), glomerulonephritis (*n* = 34), unknown (*n* = 49) and others (*n* = 44). No difference occurred regarding the distribution of underlying nephropathies between patients having anti-SARS-CoV-2 antibody in serum and those who did not (data not shown). 

As listed in Table 1, a high frequency of comorbidities has been found in our patients with CKD. Table 1 shows the background, clinical and biochemical characteristics of patients with CKD at pre-dialysis and dialysis stage (*n* = 199). There were significant differences regarding pre-dialysis versus dialysis patients with CKD with regard to some biochemical parameters and comorbidities (Table 1). No HBsAg positive patients in the study group were observed. Patients with CKD and at least three comorbidities were not more frequent in the group of anti-SARS-CoV-2-positive patients. As reported in Table 2, there was a difference concerning serum albumin levels (a marker of nutritional status) between anti-SARS-CoV-2-positive or -negative patients (*p* < 0.04). We observed no statistical relationship (univariate analysis) between positive anti-SARS-CoV-2 serology status and numerous comorbidities (arterial hypertension, diabetes mellitus, chronic obstructive pulmonary disease and others) in the entire cohort. There was no difference in the frequency of anti-SARS-CoV-2 antibody between patients on maintenance dialysis or not, 15.5% (16/103) vs. 21.6% (43/199), NS. Two (3.2%) out of 62 patients with anti-SARS-CoV-2 antibody in serum had previously been hospitalized due to SARS-CoV-2-related pneumonia. Table 3 reports the results of multivariate analysis—an independent and significant association between positive SARS-CoV-2 serologic status and serum albumin was observed (*p* < 0.046) in the entire cohort.

## 3. Discussion

The primary finding of this prospective, single-centre, observational study was the occurrence of a high frequency (20.5%) of anti-SARS-CoV-2 seropositive patients in our CKD population. Sero-prevalence rates of SARS-CoV-2 antibody vary depending on various parameters including geographic location, timing of survey, or type of population evaluated. Sero-prevalence of anti-SARS-CoV-2 antibody was 36% (129/356) among patients on long-term haemodialysis in London [10] and 5.8% (747/12,932) among maintenance dialysis patients at a large dialysis organization in the US [11]. The great prevalence of anti-SARS-CoV-2 antibody in our patient group can be explained, at least in part, by the fact that our patients were resident in Milan city, one of the cities most severely hit by SARS-CoV-2 infection. 

We observed an independent and significant link between positive serology towards SARS-CoV-2 and serum albumin in the entire cohort of patients with CKD. Total protein levels in serum were also lower in CKD patients with SARS-CoV-2 infection than in those without infection but the difference did not reach the statistical significance. It is likely that a poor nutritional status (as reflected by low serum albumin values) could support the acquisition of SARS-CoV-2 infection and consequent anti-SARS-CoV-2 antibody detection in serum. Of note, the sero-prevalence of anti-SARS-CoV-2 antibody among healthcare workers and healthy blood donors at the same hospital was 7.6% (309/4055) [12] and 5.2% (95% CI, 2.4–9.0) [13], respectively, based on two surveys performed a few months before the current survey. Anti-SARS-CoV-2 antibody detected among healthcare workers were anti-S1 and anti-S2 IgG antibodies; and healthy blood donors showed IgM/IgG antibody against the SARS-CoV-2 nucleocapsid protein. It is likely that the differences in the technologies adopted to detect antibodies clearly do not explain the results reported above. It remains unclear if the higher frequency of anti-SARS-CoV-2 seropositive status in our CKD population compared with controls in the same area is related to the presence of advanced age, several comorbidities (including poor nutritional status), or kidney impairment per se. Further factors which could increase the SARS-CoV-2 infection rate in CKD population includes uremic toxins, bone and mineral disorders, oxidative stress and endothelial dysfunction. We need further studies aimed to clarify the risk factors for anti-SARS-CoV-2 antibody acquisition. Anti-SARS-CoV-2 seropositive status was common irrespective of whether or not patients were dialysis-dependent or had advanced age. Muir and colleagues in their ESRD population (*n* = 164) found that anti-N and/or anti-S1 SARS-CoV-2 IgG antibody positive compared with antibody negative (anti-N and anti-S1) patients had a greater clinical frailty score (*p* = 0.02), and received more common haemodialysis as opposed to being pre-dialysis or peritoneal dialysis (*p* = 0.006) [14]. Wickens and colleagues found 32 (7.8%) patients with SARS-CoV-2 infection by PCR testing. The proportion of frail patients was greater in COVID-19-positive than -negative individuals (64.3% vs. 34.1%, *p* = 0.003) [15]. According to a recent systematic review with meta-analysis (14 reports, 4569 unique patients included) [16], the occurrence of many comorbidities such as hypertension, coronary heart disease and diabetes was associated with increased risk of death among patients with SARS-CoV-2 infection. 

Infections are the second leading cause of death among patients with dialysis-dependent ESRD mainly owing to the impairment of both innate and acquired immunity, related both uraemia and concomitant immunosuppression therapy [17]. Patients on maintenance dialysis are a risk-group as they have increased exposure to SARS-CoV-2 infection due to various reasons—they undergo in-centre haemodialysis (usually two or three sessions weekly) with contact with patients or dialysis staff. Additionally, risks related to transport cannot be excluded [18]. Patients on maintenance dialysis who acquire infection by SARS-CoV-2 require control and prevention and this poses significant difficulties on dialysis facilities where effective social distancing is logistically challenging. Conversely, patients receiving dialysis are less likely to be employed and more likely to have restricted social activities or mobility due to frailty or aging. Several approaches have been adopted to control nosocomial transmission of SARS-CoV-2 within dialysis facilities including lower number of patients in waiting rooms, limited shared patient transport, appropriate training of dialysis staff and regular PCR screening with nasopharyngeal swabs [18]. 

The strengths of the current study include prospective enrolment and detailed characterization of participants. Some evidence on serology for anti-SARS-CoV-2 antibody has been reported in the dialysis population [19,20,21,22] but data at pre-dialysis stage is extremely limited [5]. Serological testing is easy to obtain from patients on regular haemodialysis when they attend dialysis without the need for additional phlebotomy. 

The current study has some shortcomings. First, all seropositive anti-SARS-CoV-2 patients tested negative by nasopharyngeal swabs but the median time between serological testing and nasopharyngeal swab ranged between 7–30 days, due to logistics. In other words, the possibility of false-positive results by serology could not be excluded. On the grounds of recent studies, it has been calculated a sensitivity/specificity of 86.1%/98.9% of IgG detection test by DIA.PRO COVID-19 Serological Assay [23]. Second, the majority of patients who tested positive by anti-SARS-CoV-2 serologic assay in the current study did not undergo hospitalization due to SARS-CoV-2; thus, we evaluated a selected subset of SARS-CoV-2 infected patients. Third, we completed the study a few weeks before the initiation of the mass vaccination campaign against SARS-CoV-2 (early 2021) and this precluded an analysis on the durability of the immune response over time. Finally, we compared our study group with health care workers and blood donors attending the same hospital; it would have been better to compare our patients versus those individuals having chronic health conditions (such as cirrhosis, anaemia, etc.) and living in Milan, Italy, at the time of the first wave of SARS-CoV-2. However, evidence on this point was extremely limited. 

It is likely that new waves of SARS-CoV-2 infection will occur in the near future; thus, several strategies to reduce the risk of exposing high-risk populations (such as CKD patients) need to be taken into account. The management of CKD patients should include in-centre haemodialysis, peritoneal dialysis, telemedicine and appropriate adherence to screening and isolation guidelines. 

In conclusion, we observed a high frequency of anti-SARS-CoV-2 antibody in our cohort of patients with CKD living in Milan, one of the cities hardest hit by the pandemic in the world. Reduced serum albumin (a marker of inadequate nutritional status) was independently and significantly associated with positive anti-SARS-CoV-2 serologic status in the entire cohort of CKD patients. The prevalence of anti-SARS-CoV-2 antibody among CKD patients (20%) was greater compared with those controls who attended the same hospital over the same year. It remains to be determined whether the high frequency of anti-SARS-CoV-2 antibody in our patients is linked to kidney impairment per se or other agents. Additionally, surveys to confirm the role of poor nutritional intake in the acquisition of SARS-CoV-2 infection in the CKD population are under way. 

## 4. Methods

### 4.1. Study Design and Ethics

This was a single-centre, prospective observational study initiated on 10 August 2020. In total, 302 adult (>18 years of age) patients with chronic kidney disease were enrolled; they were on follow-up at the outpatient clinic of Maggiore Policlinico Hospital. Outpatients underwent sampling for antibody towards SARS-CoV-2 during their regular nephrology visits at Maggiore Hospital; on that occasion, patients completed a written questionnaire concerning signs/symptoms of SARS-CoV-2 infection eventually occurring within three months prior to the visit. Patients positive for anti-SARS-CoV-2 antibody underwent testing with nasopharyngeal swabs even if the median time between serological testing and nasopharyngeal swab ranged between 7–30 days, due to logistics. Demographic information, clinical presentations and laboratory tests were collected from patient medical records. The study was approved by the Ethics Committee of Maggiore Policlinico Hospital and Cà Granda IRCCS Foundation (‘COVID-19 serology study in CKD’; 27 July 2020). Each patient signed written informed consent to allow testing for communicable diseases, storage of anonymized data and biological materials, and use of data for clinical research.

### 4.2. Anti-SARS-CoV-2 Antibody Testing

DIA.PRO COVID-19 Serological Assay includes two kits for the detection of either IgG or IgM to COVID-19 and a third-module-based immunoassay for the confirmation and typing of IgG antibodies to SARS-CoV-2 major antigens. DIA.PRO COVID-19 Serological Assay is CE marked and is currently available on EIA, 96 well and microplate technology. The confirmation assay is a modular system to individually detect antibodies against COVID-19 Spike protein 1, Spike protein 2 and Nucleocapsid. Antibodies against SARS-CoV-2 in human serum and plasma have been detected. Briefly, microplates coated with recombinant antigens specific to SARS-CoV-2 are treated with diluted sample and IgG/IgM are captured, if present, by the antigens. After washing, in the second incubation phase-bound antibodies are detected by the addition of polyclonal specific anti-IgG labelled with peroxidase (HRP). The enzyme captured on the solid phase, acting on the substrate/chromogen mixture, gives an optical signal that is proportional to the amount of anti-SARS-CoV-2 IgG/IgM present in the sample. Cut-off values let optical densities be interpreted into SARS-CoV-2 IgG or IgM negative and positive results. In order to exclude false-positive results, patients tested positive by screening DIA.PRO COVID-19 Serological Assay underwent confirmatory testing with a third-module-based immunoassay (by DIA.PRO COVID-19 Serological Assay).

### 4.3. Statistical Analysis

Seroprevalence was assessed by calculating the proportion of samples considered IgM/IgG positive overall and stratified by sample collection date, gender, age and race. Descriptive statistics was summarised with mean ± standard deviation (SD) (continuous parameters); categorical variables were reported as percentages. Comparisons between groups were made with parametric and non-parametric tests, where appropriate. Results were reported according to the STROBE (Strengthening the Reporting of Molecular Epidemiology for Infectious Diseases) guidelines [24]. We performed multivariate analysis by logistic regression model; demographic, clinical and biochemical characteristics were adopted as independent variables, and a positive serologic status for anti-SARS-CoV-2 antibody was assumed as the dependent parameter. Statistical analysis was made by the program JPM (version 3.1., SAS Institute, Cary, NC, USA, 1996).

## Figures and Tables

**Table 1:** Characteristics of CKD patients at baseline.

Patients, *n*	Entire Cohort (*n* = 302)	Dialysis Independent pts (*n* = 199)	Dialysis pts (*n* = 103)	*p*
**Age, years**	71.2 ± 14.7	72.09 ± 14.5	70.3 ± 14.1	NS
**Caucasian, *n***	271 (89.7%)	182 (91.5%)	89 (86.4%)	NS
**Males, *n***	201 (66.5%)	138 (69.4%)	63 (61.2%)	NS
**Body weight, kg**	72.7 ± 18.02	76.6 ± 17.6	68.8 ± 18.4	0.001
**Azotaemia, mg/dL**	105.8 ± 48.1	82.9 ± 35.9	144.7 ± 40.9	0.001
**Serum creatinine, mg/dL**	4.57 ± 3.5	2.44 ± 1.4	6.7 ± 2.4	0.001
**Arterial hypertension, *n***	270 (89.4%)	177 (89.8%)	93 (90.3%)	NS
**Diabetes mellitus (insulin dependent), *n***	49 (16.4%)	23 (11.6%)	26 (25.2%)	0.002
**Diabetes mellitus (insulin independent), *n***	59 (19.5%)	45 (22.8%)	14 (13.6%)	NS
**Chronic obstructive pulmonary disease, *n***	40 (13.5%)	25 (12.8%)	15 (14.6%)	NS
**Chronic liver disease, *n***	60 (19.9%)	46 (23.5%)	14 (13.6%)	0.044
**Cardiovascular disease, *n***	160 (53.7%)	97 (49.4%)	63 (61.2%)	0.053
**Dyslipidaemia, *n***	162 (54.4%)	120 (61.2%)	42 (40.8%)	0.001
**Comorbidities >3, *n***	193 (63.9%)	141 (71.5%)	52 (50.5%)	0.001
**Smoke, *n***	59 (32.6%)	40 (49.4%)	19 (18.5%)	NS
**Hyperuricaemia, *n***	124 (41.5%)	104 (52.8%)	20 (19.4%)	0.001
**Serum total proteins, g/L**	6.7 ± 0.66	6.90 ± 0.8	6.39 ± 0.6	0.0001
**Serum albumin, g/L**	3.97 ± 0.43	4.06 ± 0.4	3.90 ± 0.4	0.005
**COVID-like symptoms (within three months prior the study), *n***	83 (27.5%)	48 (24.2%)	35 (33.9%)	NS
**Medical history positive for neoplastic disorders, *n***	74 (25.1%)	50 (25.9%)	24 (23.3%)	NS
**Anti-HCV antibody positive, *n***	8 (7.5%)	1 (0.5%)	7 (6.8%)	0.001

NS = not significant.

**Table 2:** Characteristics of CKD patients at baseline (anti-SARS-CoV-2-positive versus -negative).

Patients, *n*	Anti-SARS-CoV-2 pos. (*n* = 60)	Anti-SARS-CoV-2 neg. (*n* = 242)	*p*
**Age, years**	71.7 ± 14.03	70.9 ± 14.9	NS
**Caucasian, *n***	90% (*n* = 54)	89.7% (*n* = 217)	NS
**Males, *n***	63.3% (*n*=38)	68.2% (*n* = 163)	NS
**Body weight, kg**	74.29 ± 18.6	72.3 ± 17.8	NS
**Azotaemia, mg/dL**	103.1 ± 24.5	107.2 ± 36.2	NS
**Serum creatinine, mg/dL**	4.18 ± 3.3	4.67 ± 3.6	NS
**Arterial hypertension, *n***	90% (*n* = 54)	90.4% (*n* = 216)	NS
**Diabetes mellitus (insulin dependent), *n***	11.7% (*n* = 7)	17.6% (*n* = 42)	NS
**Diabetes mellitus (insulin independent), *n***	27% (*n* = 16)	17.9% (*n* = 43)	NS
**Chronic obstructive pulmonary disease, *n***	11.9% (*n* = 7)	13.9% (*n* = 33)	NS
**Chronic liver disease, *n***	21.7% (*n* = 13)	19.7% (*n* = 47)	NS
**Cardiovascular disease, *n***	58.3% (*n* = 35)	52.5% (*n* = 125)	NS
**Dyslipidaemia, *n***	53.3% (*n* = 32)	54.6% (*n* = 130)	NS
**Comorbidities** **≥** **3, *n***	68.3% (*n* = 41)	62.8% (*n* = 152)	NS
**Smoke, *n***	43.2% (*n* = 16)	29.9% (*n* = 43)	NS
**Hyperuricaemia, *n***	45% (*n* = 27)	40.6% (*n* = 97)	NS
**Serum total proteins, g/L**	6.63 ± 0.85	6.73 ± 0.60	NS
**Serum albumin, g/L**	3.84 ± 0.51	4.0 ± 0.43	0.042
**COVID-like symptoms (within three months prior the study), *n***	33.9% (*n* = 21)	25.9% (*n* = 62)	NS
**Medical history positive for malignancy, *n***	25% (*n* = 15)	25.2% (*n* = 59)	NS
**Anti-HCV antibody positive, *n***	0	8.9% (*n* = 8)	NS

NS = not significant.

**Table 3:** Multivariate analysis: parameter estimates and effect test (dependent parameter: positive serologic status for anti-SARS-CoV-2 antibody) (*n* = 302 patients).

Effect Test	B	SE	Wald chi^2^	DF	Exp (B)	*p*
**COPD**	−0.134	0.443	0.092	1	0.874	0.762
**CLD**	−0.331	0.414	0.639	1	0.718	0.424
**CVD**	0.001	0.325	0.000	1	1.001	0.997
**Dyslipidaemia**	0.129	0.329	0.153	1	1.137	0.695
**DM ID**	0.071	0.409	0.03	1	1.074	0.862
**DM non-ID**	0.687	0.413	2.773	1	1.989	0.096
**Kidney status**	−0.450	0.326	1.912	1	0.637	0.167
**Albumin**	−0.711	0.356	3.996	1	0.491	0.046
**Constant**	2.029	1.473	1.896	1	7.604	0.169

## Abbreviations

**AH**	Arterial hypertension
**AKI**	Acute kidney injury
**APKD**	Adult polycystic kidney disease
**CI**	Confidence intervals
**CKD**	Chronic kidney disease
**COVID-19**	Coronavirus disease 2019
**CLD**	Chronic liver disease
**COPD**	Chronic obstructive pulmonary disease
**CVD**	Cardiovascular disease
**DM**	Diabetes mellitus
**eGFR**	Estimated glomerular filtration rate
**ESRD**	End-stage renal disease
**HBsAg**	Hepatitis B virus antigen
**HCV**	Hepatitis C virus
**HCW**	Health care worker
**HD**	Haemodialysis
**KDIGO**	Kidney Disease: Improving Global Outcomes
**NA**	Not available
**RRT**	Renal replacement therapy
**SARS-CoV-2**	Severe acute respiratory syndrome coronavirus 2
**WHO**	World health organization
